# Combined Use of Multiple Intravascular Imaging Techniques in Acute Coronary Syndrome

**DOI:** 10.3389/fcvm.2021.824128

**Published:** 2022-01-17

**Authors:** Takashi Kubo, Kosei Terada, Yasushi Ino, Yasutsugu Shiono, Shengxian Tu, Tien-Ping Tsao, Yundai Chen, Duk-Woo Park

**Affiliations:** ^1^Department of Cardiovascular Medicine, Wakayama Medical University, Wakayama, Japan; ^2^Department of Cardiovascular Medicine, Naga Municipal Hospital, Kinokawa, Japan; ^3^Department of Cardiovascular Medicine, Shingu Municipal Hospital, Shingu, Japan; ^4^School of Biomedical Engineering, Biomedical Instrument Institute, Shanghai Jiao Tong University, Shanghai, China; ^5^Division of Cardiology, Heart Center, Cheng Hsin General Hospital, Taipei, Taiwan; ^6^Department of Cardiology, Chinese PLA General Hospital, Beijing, China; ^7^Division of Cardiology, Asan Medical Center, University of Ulsan College of Medicine, Seoul, South Korea

**Keywords:** acute coronary syndrome, plaque rupture, plaque erosion, calcified nodule, intravascular ultrasound, optical coherence tomography, near-infrared spectroscopy

## Abstract

Recent advances in intravascular imaging techniques have made it possible to assess the culprit lesions of acute coronary syndrome (ACS) in the clinical setting. Intravascular ultrasound (IVUS) is the most commonly used intravascular imaging technique that provides cross-sectional images of coronary arteries. IVUS can assess plaque burden and vessel remodeling. Optical coherence tomography (OCT) is a high-resolution (10 μm) intravascular imaging technique that uses near-infrared light. OCT can identify key features of atheroma, such as lipid core and thin fibrous cap. Near-infrared spectroscopy (NIRS) can detect lipid composition by analyzing the near-infrared absorption properties of coronary plaques. NIRS provides a chemogram of the coronary artery wall, which allows for specific quantification of lipid accumulation. These intravascular imaging techniques can depict histological features of plaque rupture, plaque erosion, and calcified nodule in ACS culprit lesions. However, no single imaging technique is perfect and each has its respective strengths and limitations. In this review, we summarize the implications of combined use of multiple intravascular imaging techniques to assess the pathology of ACS and guide lesion-specific treatment.

## Introduction

Several autopsy studies have revealed the pathology of culprit lesions of acute coronary syndrome (ACS). ACS is caused by three main mechanisms: plaque rupture, plaque erosion, and calcified nodule. Plaque rupture is the most common (55–60%) mechanism for ACS (1). Plaque rupture usually has an extensive lipid core containing large numbers of cholesterol crystals and a thin fibrous cap (<65 μm) infiltrated by foamy macrophages. Plaque erosion is found in 30–35% of ACS (1). Most of plaque erosion occurs over areas of intimal thickening, with minimal or no evidence of a lipid. Calcified nodule is identified in 2–7% of ACS (1). Calcified nodule is a plaque with luminal thrombi showing calcific nodules protruding into the lumen through a disrupted thin fibrous cap.

Recent advances in intravascular imaging techniques have made it possible to assess the culprit lesions of ACS in the clinical setting. In the early 1990s, intravascular ultrasound (IVUS) became clinically available, enabling morphological evaluation of coronary artery walls. In the mid-2000s, optical coherence tomography (OCT) was developed and its high resolution allows for more precise observations of coronary atherosclerosis. In the late 2000s, near-infrared spectroscopy (NIRS) was clinically applied, which permits to diagnose plaque composition. These intravascular imaging techniques can depict histological features of plaque rupture, plaque erosion, and calcified nodule in ACS culprit lesions (2).

## IVUS

IVUS is the most commonly used intravascular imaging technique in the world. IVUS provides cross-sectional images of coronary arteries. IVUS can identify plaque rupture and calcified nodule, but not plaque erosion. Plaque rupture is identified as ulceration with a recess in the plaque beginning at the luminal-intimal border (3, 4). The lesions with plaque rupture usually have a large plaque burden with positive vascular remodeling and exhibit a hypoechoic plaque with acoustic shadow derived from the lipid core (so-called attenuated plaque) (5). The coarse-resolution (100–200 μm) of IVUS cannot determine the presence or absence of small rupture, therefore this technique cannot diagnose plaque erosion. Calcified nodule is identified by a convex shape of the luminal surface with a bright echo, bulgy shape, irregular surface, and acoustic shadowing (6). The lesions with calcified nodule often have an extensive calcium sheet.

## OCT

OCT is a high-resolution (10 μm) intravascular imaging technique that uses near-infrared light. OCT is the most reliable technique for evaluating the complex morphology of ACS culprit lesions, although this technique requires some complicated procedures such as intracoronary contrast injection to remove red blood cells for image acquisition. OCT can identify lipid core (characterized by signal-poor regions with diffuse borders), thin fibrous cap (characterized by homogeneous, signal-rich regions), and calcification (characterized by well-delineated, signal-poor regions with sharp borders) in atherosclerotic plaques (7). Plaque rupture is defined as a disruption of thin fibrous cap with a clear cavity formed inside the plaque (8). The lesions with plaque rupture often have macrophages (defined by signal-rich, distinct or confluent punctuate regions with shadowing), vasa vasorums (defined by signal-poor, well-delineated voids within plaque), cholesterol crystals (defined as thin, linear regions of high signal intensity within the lipid plaque), and healed plaques (defined as plaques with 1 or more layers with different optical density) (9, 10). Plaque erosion is identified by the presence of attached thrombus overlying an intact (i.e. absence of fibrous cap disruption) plaque without superficial lipid or calcification (11). The absence of endothelial cells is a key pathological criterion for erosion, however the resolution of current OCT cannot detect individual endothelial cells. Since the OCT metrics of plaque erosion are different from the pathological definition, the term “OCT-derived erosion” is used instead of erosion. Calcified nodule is defined by fibrous cap disruption detected over a calcified plaque characterized by protruding calcification (11). Most of calcified nodule have superficially located large calcification. In ACS culprit lesions, a large amount of thrombus may cause a strong attenuation of the near-infrared light and interfere with accurate OCT assessment of plaque morphology.

## NIRS

NIRS can identify lipid composition by analyzing the near-infrared absorption properties of coronary plaques. NIRS provides a chemogram of the coronary artery wall, which enables the detection of lipid core and specific quantification of lipid accumulation measured as the lipid core burden index (LCBI) and maximal LCBI over any 4 mm segment (maxLCBI4mm). Unlike IVUS and OCT, NIRS can accurately measure lipid component even in the presence of thrombi. NIRS is currently available as a combination catheter with IVUS. NIRS can compensate for the lack of IVUS ability to detect plaque erosion. Plaque erosion has a significantly lower maxLCBI4mm than plaque rupture and calcified nodule (12, 13). The optimal cutoff value for maxLCBI4mm to differentiate between plaque erosion and other plaque types is ~400 (12). By assessing plaque cavity, convex calcium, and maxLCBI4mm, NIRS-IVUS can accurately identify plaque rupture (sensitivity = 97% and specificity = 96%), plaque erosion (sensitivity = 93% and specificity = 99%), and calcified nodule (sensitivity = 100% and specificity = 99%) (13).

## Multimodality Imaging

**Figure 1:**
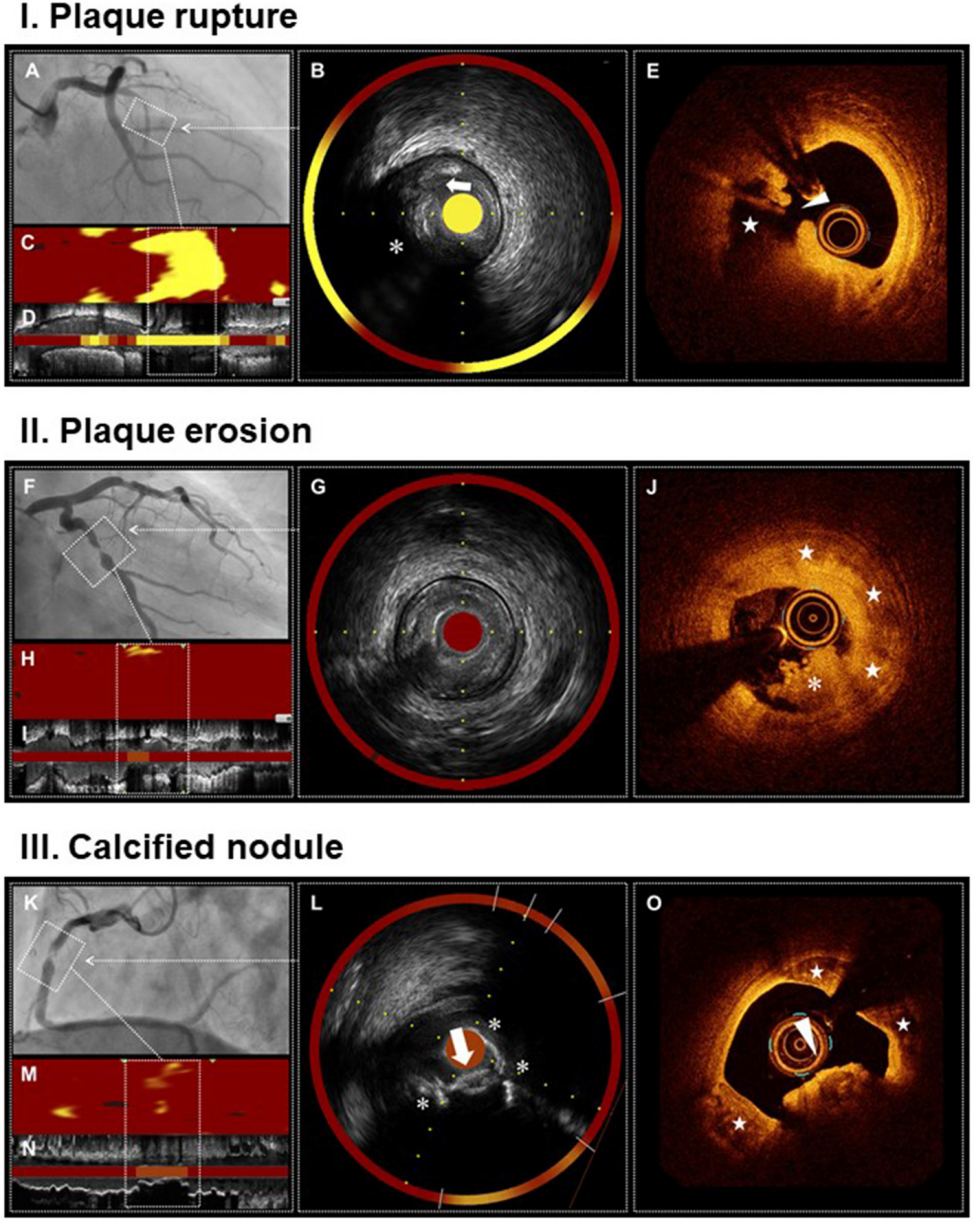
Multimodality imaging of plaque rupture, plaque erosion, and calcified nodule. Plaque rupture (I) Angiogram shows occlusion of proximal LAD, the culprit lesion of STEMI **(A)**. IVUS demonstrates ulceration (arrow) with a recess on the surface of attenuated plaque (asterisk) **(B,D)**. NIRS identifies a large lipid content (maxLCBI4mm = 920) **(B–D)**. OCT reveals plaque rupture characterized by a fibrous cap disruption (arrowhead) and a cavity (star) formation inside the plaque **(E)**. Plaque erosion (II) Angiogram shows severe stenosis in mid LCX, the culprit lesion of STEMI **(F)**. IVUS demonstrates the absence of plaque ulceration **(G,I)**. NIRS identifies a small lipid content (maxLCBI4mm = 129) **(G–I)**. OCT reveals plaque erosion characterized by the presence of attached thrombus (asterisk) overlying an intact fibrotic plaque (stars) **(J)**. Calcified nodule (II) Angiogram shows severe stenosis and intraluminal filling defect in mid RCA, the culprit lesion of non-STEMI **(K)**. IVUS demonstrates a convex calcium with irregular surface (arrow) and large superficial calcium (asterisks) **(L,N)**. NIRS identifies a moderate lipid content (maxLCBI4mm = 208) **(L–N)**. OCT reveals calcified nodule characterized by a protruding calcium with thrombi (arrowhead) and large superficial calcium (stars) **(O)**. IVUS, intravascular ultrasound; LAD, left anterior descending artery; LCBI, lipid core burden index; LCX, left circumflex artery; NIRS, near-infrared spectroscopy; OCT, optical coherence tomography; RCA, right coronary artery; STEMI, ST-segment elevation myocardial infarction.

Information obtained from multiple imaging techniques can complement each other. In fact, in addition to morphological evaluation, plaque tissue characterization is useful for differentiating plaque rupture, plaque erosion, and calcified nodule (Table 1). Plaque rupture is identified by fibrous cap disruption and intra-plaque cavity in IVUS/OCT, attenuated plaque in IVUS, large lipid plaque in OCT, and high maxLCBI4mm (>400) in NIRS (Figure 1-I). Plaque erosion is identified by intact fibrous cap and fibrotic plaque in OCT, and low maxLCBI4mm (<400) in NIRS (Figure 1-II). Calcified nodule is identified by convex calcium and large calcium sheet in IVUS/OCT, and intermediate maxLCBI4mm in NIRS (Figure 1-III).

**Table 1:** Diagnostic criteria for plaque rupture, erosion, and calcified nodules.

	**IVUS**	**OCT**	**NIRS**
Plaque rupture	Fibrous cap disruption Intra-plaque cavity Attenuated plaque	Fibrous cap disruption Intra-plaque cavity Large lipid plaque Macrophages Vasa vasorum Cholesterol crystals Healed plaque	High maxLCBI 4mm (>400)
Plaque erosion	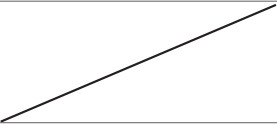	Intact fibrous cap Fibrotic plaque	Low maxLCBI 4mm (<400)
Calcified nodule	Convex calcium Large calcium sheet	Convex calcium Large calcium sheet	Moderate maxLCBI 4mm

*IVUS, intravascular ultrasound; LCBI, lipid core burden index; NIRS, near-infrared spectroscopy; OCT, optical coherence tomography*.

## Impact on Treatment Strategy

Information from multiple imaging techniques influence percutaneous coronary intervention (PCI) strategies and risk stratification in ACS. Plaque rupture is at high risk of slow flow or no-reflow, distal embolization, and microvascular obstruction associated with PCI (14–16). This is because the lesions with plaque rupture are rich in thrombi and lipid cores that can be embolic sources. A prospective randomized controlled trial using IVUS showed that the use of distal protection devise was effective in preventing slow flow during PCI for attenuated plaques (17). Plaque erosion has a better prognosis after PCI compared with plaque rupture (18, 19). Non-stent strategy may be an option for plaque erosion if sufficient lumen is obtained by thrombus aspiration alone. A prospective observational OCT study demonstrated that a majority (92.5%) of patients with ACS caused by plaque erosion managed with dual antiplatelet therapy without stenting remained free of major adverse cardiovascular event during 1-year follow-up period (20). Calcified nodule is associated with stent underexpansion that is a well-known predictor of poor clinical outcome. An OCT-guided PCI registry disclosed that the minimum stent area at post-PCI was significantly smaller in calcified nodule than in plaque rupture and plaque erosion, with half of calcified nodule having a stent expansion index (calculated by minimum stent area / reference lumen area x 100) of <80% (21). Further research is needed to evaluate the effectiveness of more aggressive procedures such as rotational atherectomy, excimer laser coronary angioplasty, and shockwave intravascular lithotripsy for calcified nodules.

## Limitations

Each intravascular imaging technique has its inherent limitations. IVUS has a coarse-resolution that is insufficient to detect ruptured thin fibrous caps and small ulcers on plaque surface. OCT has a small field of view and shallow imaging depth that often preclude assessment of the plaque burden and remodeling pattern. NIRS is unable to identify fibrous caps, in addition to its lack of morphological evaluation ability.

## Future Perspectives

To overcome the limitations of each imaging technique, new efforts have been developed for data fusion methodologies and designs for hybrid dual-probe catheters. Combined IVUS-OCT imaging provides a fusional image of OCT with a high resolution for the plaque surface and IVUS with a large imaging depth (22). Combined IVUS-OCT imaging enables a comprehensive depiction of coronary atherosclerotic plaque. Combined NIRS-OCT imaging allows the detection of lipid core by spectroscopy and structural features, including cap thickness, by OCT (23). Combined NIRS-OCT imaging will facilitate the identification of thin-capped fibroatheroma, which is recognized as a precursor to plaque rupture.

## Conclusion

At this time, there is no single imaging technique that is perfect for evaluating atherosclerosis. The combination of multiple imaging techniques provides a lot of information about the morphology and composition of atherosclerosis. Detailed coronary evaluation with multiple imaging techniques allows a better understanding of the pathology of ACS and guides lesion-specific treatment.

## Author Contributions

TK wrote the manuscript. KT, YI, and YS collected images of IVUS, OCT, and NIRS. ST, T-PT, YC, and D-WP reviewed and revised the manuscript. All authors approved the final manuscript.

## Funding

This work was partially supported by JSPS KAKENHI Grant Number JP19K12846.

## Conflict of Interest

The authors declare that the research was conducted in the absence of any commercial or financial relationships that could be construed as a potential conflict of interest.

## Publisher's Note

All claims expressed in this article are solely those of the authors and do not necessarily represent those of their affiliated organizations, or those of the publisher, the editors and the reviewers. Any product that may be evaluated in this article, or claim that may be made by its manufacturer, is not guaranteed or endorsed by the publisher.
